# 

**Published:** 1874-04

**Authors:** 


### CONTENTS:

ORIGINAL COMMUNICATIONS:

ARTICLE I.—Valedictory Address to the Graduating Class, Rush Med. College, Feb. 17, 1874. By De Laskie Miller, M.D. ..... 193

ART. II.—Myelitis. By Walter Hay, M.D. ..... 207

ART. III.—A Case of Paraplegia from Peripheral Irritation. By G. W. Stewart, M.D. ..... 209

ART. IV.—Case of Monstrosity. By F. E. English, M.D. ..... 213

PROGRESS IN MEDICAL SCIENCES:

ART. I.—Neuro Pathology and Psychological Medicine. By Walter Hay, M.D. ..... 215

ART. II.—Report on Obstetrics and Gynæcology. By A. Reeves Jackson, M.D. ..... 220

ART. III.—Progress of Surgery. By J. E. Owens, M.D. ..... 224

HOSPITAL REPORTS ..... 228

CLINICS ..... 233

REPORTS OF SOCIETIES ..... 237

EDITORS' BOOK TABLE ..... 246

EDITORIAL ..... 248

CHICAGO MORTALITY REPORT, FOR FEB. 1874 ..... 254

